# Effect of Shoulder Movement Routine on Postoperative Shoulder Pain in Total Laparoscopic Hysterectomy: A Randomized Clinical Trial

**DOI:** 10.3390/medicina60091478

**Published:** 2024-09-10

**Authors:** Andrea Olguín-Ortega, Lino Palacios-Cruz, Alejandro Rendón-Molina, Oliver Cruz-Orozco, Brenda Sánchez-Ramírez, Silvia Fabiola Estrada-Rivera, José Roberto Silvestri-Tomassoni, Ana Cristina Arteaga-Gómez, Enrique Reyes-Muñoz

**Affiliations:** 1 Department of Gynecology, Instituto Nacional de Perinatología Isidro Espinosa de los Reyes, Montes Urales 800, Mexico City 11000, Mexico; olguin.andrea@gmail.com (A.O.-O.); arendonm.87@gmail.com (A.R.-M.); oliverpco@gmail.com (O.C.-O.); dra.bsr@gmail.com (B.S.-R.); dra.estrada.oncogine@gmail.com (S.F.E.-R.); dr.silvestri6511@gmail.com (J.R.S.-T.); 2 Facultad de Ciencias de la Salud, Universidad Anáhuac México, Campus Norte, Av. Universidad Anáhuac 46, Huixquilucan 52786, Mexico; 3 Department of Clinical Epidemiology, Instituto Nacional de Psiquiatría Dr. Ramón de la Fuente Muñiz, Calzada Mexico-Xochimilco 101, Mexico City 14370, Mexico; palacioslino@gmail.com; 4 General Direction, Instituto Nacional de Perinatología Isidro Espinosa de los Reyes, Montes Urales 800, Mexico City 11000, Mexico; acaarteaga@yahoo.com.mx; 5 Research Division, Instituto Nacional de Perinatología Isidro Espinosa de los Reyes, Montes Urales 800, Mexico City 11000, Mexico

**Keywords:** laparoscopy hysterectomy, shoulder pain, postoperative pain, shoulder movement

## Abstract

*Background and Objectives:* Postoperative shoulder pain is a common issue after total laparoscopic hysterectomy (TLH). This study evaluated the impact of a shoulder movement routine on postoperative shoulder pain in women undergoing uncomplicated TLH. *Materials and Methods:* An open-label randomized clinical trial included women without prior shoulder pain undergoing TLH between 20 January and 20 March 2024. Participants were randomized into two groups: Group 1 (*n* = 36) received a shoulder movement routine, while Group 2 (control, *n* = 39) performed a hand movement routine. Shoulder pain was assessed using the visual analog scale (VAS) at 6 h, 24 h, and 7 days postoperatively. *Results:* Seventy-five women participated. No significant differences were found between the groups regarding demographic variables, surgery duration, or hospital stay. Shoulder pain scores (VAS) at three time points (6 h, 24 h, and 7 days) showed no significant differences between groups (*p* = 0.57, *p* = 0.69, and *p* = 0.91, respectively). Similarly, there were no significant differences in incisional or abdominal pain. *Conclusions:* The shoulder movement routine did not significantly reduce postoperative shoulder pain in women undergoing uncomplicated TLH.

## 1. Introduction

Hysterectomy is the most performed gynecological surgery worldwide [1]. Total laparoscopic hysterectomy (TLH) remains the foremost surgical option for benign gynecological conditions in Taiwan and around the world [2]. Following a laparoscopy, up to 80% of patients may experience shoulder pain, potentially resulting in extended hospital stays and increased risk of readmission; consequently, specific techniques are being implemented during gynecological procedures to mitigate the prevalence and severity of such pain, but several interventions require to be evaluated through randomized controlled trials in the context of gynecological laparoscopy [3].

TLH induces various postoperative pain types, including generalized, visceral, incisional, shoulder, and perineal pain. Visceral and incisional pain peaked immediately post-surgery, while shoulder pain intensified gradually, peaking at 24 h and becoming the most severe [4]. About 30% of patients experience moderate to severe postoperative pain; early identification of at-risk individuals could advance personalized pain management, improving outcomes through tailored treatments based on specific pain mechanisms [5]. Postoperative shoulder pain is a discomfort commonly attributed to carbon dioxide-related peritoneal distension and diaphragmatic irritation, with studies showing a clear correlation between the volume of residual gas and postoperative pain intensity [6].

Rapid peritoneal inflation during laparoscopy can cause vascular rupture, nerve stretching, and inflammatory release, leading to shoulder pain via phrenic nerve irritation. Both laparotomy and laparoscopy are associated with prolonged pain due to pneumoperitoneum, which can last up to three days; however, gas evacuation beneath the diaphragm can alleviate this discomfort [7]. A meta-analysis encompassing five randomized clinical trials with a total of 367 subjects demonstrated that active gas aspiration substantially decreases residual gas volumes and analgesic requirements compared to passive aspiration, significantly reducing shoulder pain scores 24 h after surgery, although it did not affect the duration of hospital stay or abdominal pain scores [8]. Multiple studies have been carried out in postoperative laparoscopy patients in which physical maneuvers were applied, including breathing and lung recruitment exercises in the postoperative period, to mitigate shoulder pain and reduce the use of rescue analgesics [9,10,11]. A study showed that abdominal compression and pulmonary recruitment significantly reduced residual pneumoperitoneum and post-laparoscopic shoulder pain after transvaginal NOTES, with patients reporting less shoulder pain than controls at 24 and 48 h, underscoring the effectiveness of these interventions in pain management [12].

A study regarding the effect of acute isometric, aerobic, or dynamic resistance exercise on pain threshold revealed that all three exercise forms diminished the sensation of experimentally induced pain in healthy individuals to varying degrees, contingent upon the pain induction technique and exercise regimen utilized [13]. Isometric exercise has hypoalgesic effects, which are not confined to the active body part but extend distally, potentially due to the modulation of nociceptive transmission through spinal gating and top-down descending pain inhibition mechanisms [14]. Also, acupressure therapy on collateral meridians, similar to shiatsu, has been used for the treatment of pain in the tip of the shoulder after laparoscopic cholecystectomy, showing in both cases a notable relief of pain and a decrease in the temperature of skin (one degree Celsius) on the affected shoulder, significantly reducing patients’ pain scores immediately after therapy [15]. In another study, the Trendelenburg position significantly reduced postoperative shoulder pain at 12 h in patients following gynecologic laparoscopic surgery, making it an effective non-pharmacologic intervention [16].

In our institution and other Mexican hospitals, 80% of women undergoing TLH are prescribed shoulder movement routines to reduce postoperative pain; however, randomized clinical trial evidence on its effectiveness is limited. We hypothesize that the exercise’s hypoalgesic effect and intra-abdominal CO_2_ redistribution may help reduce referred shoulder pain. This study aims to evaluate the impact of a shoulder movement routine compared to a hand movement routine (as a control group) on reducing postoperative shoulder pain in women who have undergone TLH. This is the first study of its kind in the laparoscopy field.

## 2. Materials and Methods

### 2.1. Participants

This open-label randomized clinical trial was approved by the Ethics and Research Internal Review Board of the Instituto Nacional de Perinatología Isidro Espinosa de los Reyes in Mexico City (Registry number: 2022-1-40, 16 March 2023) and was registered in clinicaltrial.gov Identifier NCT06195176, where the full trial protocol can be accessed. All participants signed written informed consent. We included women undergoing uncomplicated TLH in our institution from 20 January 2024 to 20 March 2024. Inclusion criteria were women aged >18, an indication of TLH for benign gynecologic disease, and without shoulder pain before surgery. Exclusion criteria were women with complications that required admission to the intensive therapy unit, surgical re-intervention, and women with a request to abandon the study; as well as those that were prescribed an analgesic infuser postoperatively.

### 2.2. Outcomes

The primary outcome of this study was to evaluate the effectiveness of a shoulder movement routine versus a hand movement routine to decrease shoulder pain intensity utilizing a standardized visual analog scale (VAS) at 6 h, 24 h, and 7 days after TLH. The VAS is a linear tool with endpoints representing “no pain” and “the worst pain imaginable”, on which patients indicate their pain level [17]. This visual analog scale (VAS) employed a numerical range from 0 to 10, supplemented by color-coded facial expressions, to quantify the subjective experience of pain from “No Pain” (0) to “Worst Pain Possible” (10).

The secondary outcome was to compare the incisional and abdominal pain between groups using the VAS.

### 2.3. Procedure

**Figure 1:**
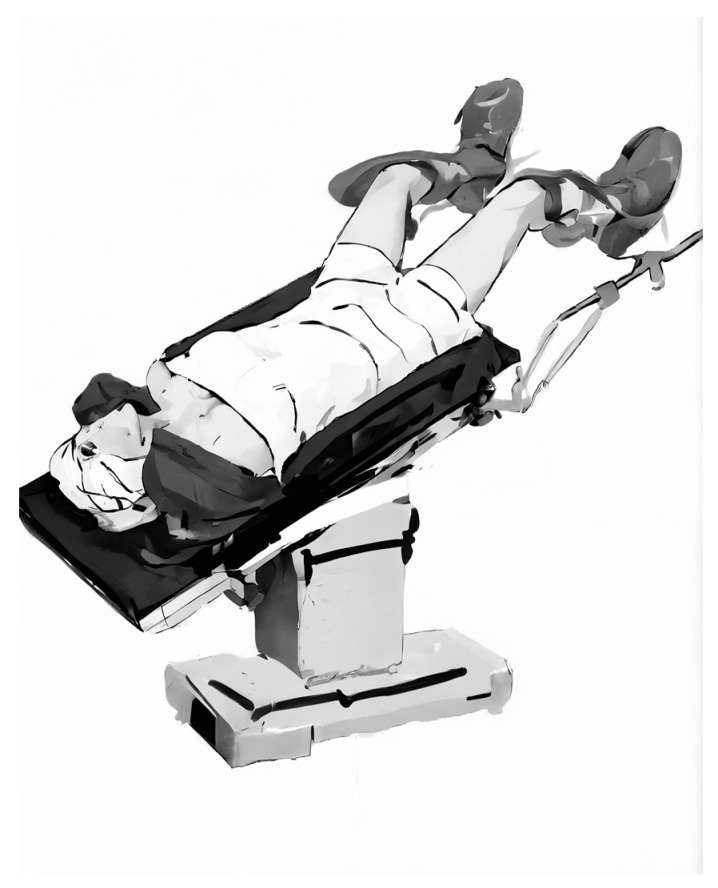
Graphic representation of the patient’s position on the surgical table.

All women scheduled for TLH for benign conditions in the morning were considered so that the movement routine had the same approximate duration in all patients during the postoperative period. All participants were placed with shoulder pads on the surgical table, a hip angulation of 15 degrees with pneumatic legs, and an intra-abdominal pressure of 15 mmHg or less during the surgical procedure (Figure 1). The ancillary trocars were not placed in the same sites for all surgeries, as their placement varied according to each surgeon’s preference. The indications for TLH for benign conditions included myoma uteri, adenomyosis, endometriosis, abnormal uterine bleeding (AUB) resistant to medical treatment, and endometrial and cervical premalignant lesions. The surgical steps adhered to a conventional approach as described previously in the literature [18]. Uniformity was maintained across all procedures by employing the same laparoscopy tower brand, ensuring the operative setup’s consistency. All the patients had mixed-type anesthesia (general anesthesia and epidural block). During the surgeries, bipolar advanced energy devices were exclusively used, except colpotomy, which was performed using a monopolar spatula. The surgeries were performed with Lopez-Zepeda (Guadalajara, Mexico) and RUMI (Cooper Surgical, Trumbull, CT, USA) uterine manipulators, which helped to provide optimal visualization and maneuverability within the pelvic cavity. None of the patients had an analgesic infuser after the surgery.

The principal investigator was blinded to the randomization group, and another investigator evaluated the participants. As this was an open-label randomized study, no one was blinded, given the nature of the intervention. The lead author generated the random allocation sequence. Randomization involved the use of a box containing opaque, sealed envelopes, with each envelope indicating the assigned intervention group. An equal number of envelopes for both groups were prepared and shuffled. They were segmented into blocks of 10, and upon completion of one block, another was used until all 78 were completed. The patient selected the corresponding envelope. The box with the envelopes was safeguarded by the principal investigator. Patients randomized to the movement routine were instructed on the movement regimen in the recovery room, and the investigator who enrolled the patient ensured the correct performance of the movements in the immediate postoperative period. After that, adherence to the movements was self-reported by each participant. The self-monitoring sheets were collected by the person who enrolled the patient in the study 7 days post-procedure at their follow-up appointment. One of the investigators enrolled patients who were candidates for the study.

### 2.4. Maneuver

The participants were allocated to one of two groups. Group 1 was assigned to execute a shoulder movement routine, which entailed elevating their shoulders toward the ears, sustaining this posture for a triad of seconds, followed by a period of rest. This sequence was mandated to be replicated tenfold at the commencement of each hour during the initial postoperative stage, with a nocturnal hiatus. Group 2 (control) was assigned to execute a hand movement routine that required the participants to alternately clench and unclench their fingers into a fist, maintaining the clenched position for a similar duration of three seconds before relaxing, with the same frequency and suspension of routine as Group 1 but without a shoulder movement (the control group did not execute the shoulder movement routine).

The movement routine was monitored only in the initial series when the women were instructed on how to execute them. Reminders were made in person, and the pain in the shoulders, incisions, and abdomen were evaluated at 6 h and 24 h postoperatively, as well as at the seven-day follow-up consultation. Patients were instructed to rate the pain at rest. The patients engaged in the prescribed movements exclusively during their in-hospital stay, limiting their activity to daytime hours. All patients were encouraged to perform their movements while hospitalized, which typically spans approximately 24 h post-surgery.

If the patient was discharged in less than 24 h following their surgery, they were instructed to fill out the self-monitoring sheet. In instances where patients’ hospital stays were prolonged, they were instructed to continue the movements until their discharge, which was still restricted to daytime hours only. The duration of hospitalization and the number of movement routines completed were recorded as part of the study outcomes. The analgesic scheme was the usual scheme used, recorded in the patient’s records in both groups, and the use of rescue analgesic (tramadol) in case the patient persisted with severe pain was also recorded. The usual analgesia regimen in our hospital consists of acetaminophen every 8 h alternated with a nonsteroidal anti-inflammatory drug (NSAID), most commonly ketorolac in 90% of cases; the same analgesic regimen was recommended during five to seven days in the postoperative period. Participants were instructed to visit the emergency department if their pain did not subside with this regimen.

The primary outcome of this study was shoulder postoperative pain at 6 h. A data recording sheet was used, and the participant wrote down a visual analog scale (VAS) from 0 to 10, a graphic representation of the pain in the shoulders, incisions, and abdominal pain at 6 and 24 h, and on the seventh day post-procedure. The trial concluded once the sample size was fully enrolled.

The data collection sheet included the schedule and dosage of analgesics, whether any rescue analgesics were required, and the patient’s self-recorded adherence to the movement routine. Potential harm was systematically evaluated using participants’ self-reports, and no instances of harm were reported by any of the groups.

### 2.5. Sample Size Calculation

Through a formula for the difference of means of independent samples, taking into account a VAS reported at 6 h post-surgery with a mean in the experimental group of 3.0 (standard deviation (SD) ± 2.0) and the control group of 4.5 (SD ± 2.0), with a two-tailed hypothesis, statistical power of 80%, and an alpha error of 0.05%, the study required sample size of 28 participants per group plus 15% of possible loss to follow-up; a total of 33 participants were required for each group [19]. It should be noted that there were no prior studies, so a pilot involving ten patients was used as a reference point with a mean reported shoulder pain VAS at 6 h of 3.0 ± 2.0.

### 2.6. Statistical Analysis

Statistical analysis was performed according to the CONSORT 2010 recommended guidelines for reporting parallel-group randomized trials [19].

The qualitative variables were described using frequencies and percentages, and the quantitative variables were described using mean, standard deviation, and interquartile range. The Shapiro–Wilk test was conducted to determine the normality of each quantitative variable, which resulted in a non-normal distribution; a chi-square test or Fisher’s exact test was used for differences in proportions, and the Mann−Whitney U test was used for mean differences. Statistical analysis was performed using SPSS version 24 (Chicago, IL, USA).

## 3. Results

**Figure 2:**
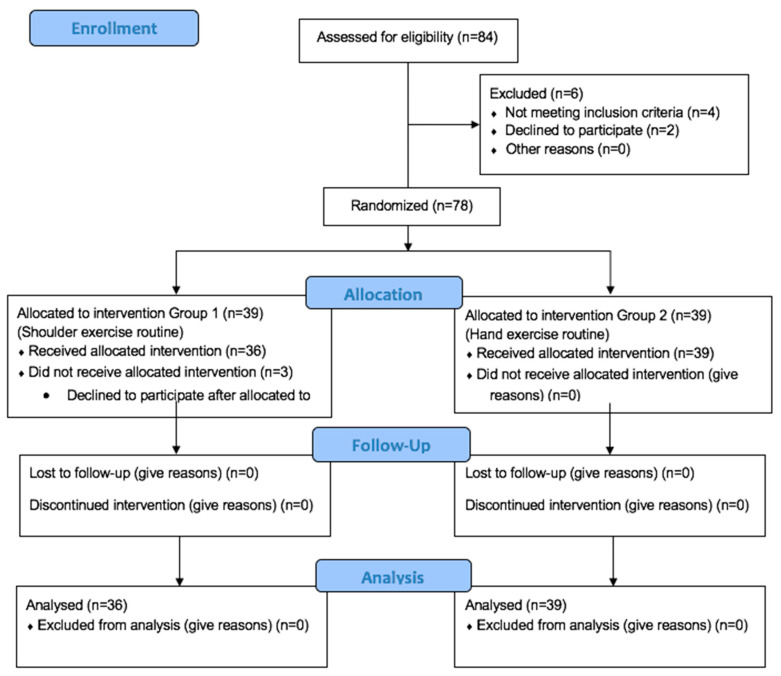
Flow chart of participants according to CONSORT guidelines.

A total of 84 women were eligible to participate in this study, and 75 received the allocated intervention (Figure 2) Group 1, shoulder movement routine *n* = 36, and Group 2, hand movement routine *n* = 39. The median age was 41 (IQR 38–47 years), with a body mass index of 29.5 (IQR 26.3–31.5 kg/m^2^). The median duration of HTL surgery was 130 (IQR 100–170 min), with a bleeding of 150 (IQR 80–200 mL), with the use of four laparoscopic ports (IQR 3–4). On average, the patients stayed hospitalized postoperatively for 24 h (IQR 21–27) and performed nine (IQR 7–10) movement routines assigned in the postoperative period. Only 11 patients in the study did not report shoulder pain; 6 were in Group 1 and 5 in Group 2 (*p* = 0.85); of these 11 patients, none required extra analgesic.

Table 1 shows the demographic characteristics of the participants. There were no significant differences in age, body mass index, average duration of surgery, CO_2_ pressure used, bleeding amount, number of trocars used, preoperative hemoglobin, systolic or diastolic blood pressure, heart rate, respiratory rate, rescue analgesia, or postoperative time, which were comparable between the two groups. Rescue analgesia consisted of an extra dose of tramadol.

Table 2 illustrates a comparative analysis of pain outcomes between the two groups assessed via the visual analog scale at varying postoperative intervals: immediately (0 h) and at 6 h, 24 h, and 7 days. The analysis revealed no statistically significant differences between groups in pain scores across all evaluated time points, as indicated by Mann–Whitney U test results (*p* > 0.05). Both groups reported a progressive decline in pain from initial measurements, with all types of pain (shoulder, incision, and abdominal) abating to zero by the seventh day.

In Table 3, the shoulder movement group, we observed a statistically significant reduction in pain across three distinct categories: shoulder pain, incision pain, and abdominal pain, assessed at intervals of 6 h, 24 h, and 7 days post-procedure. Initially, all the patients were pain free; afterward, median shoulder pain at 6 h was reported at 3 (IQR 2–5), which significantly decreased to 1 (IQR 0–4) at 24 h, and resolved entirely by 7 days (0 IQR 0–0). Similar patterns were noted in incision and abdominal pain. Statistical analysis employing the Friedman test with post hoc Wilcoxon signed-rank tests confirmed the significance of these reductions (*p* = 0.001 for all comparisons).

Table 4 presents the median VAS at multiple time points post-intervention: at 6 h, 24 h, and 7 days in the hand movement group. Significant reductions in pain were observed across all measured categories—shoulder pain, incisional pain, and abdominal pain—with initial median scores decreasing from moderate levels to zero by the seventh day.

## 4. Discussion

In our population, 85.5% of patients presented shoulder pain 6 h after the procedure, which agrees with the world literature that reported from 68% to 90% [3,20]. Other studies have reported that more than 90% of patients experience shoulder pain on the first day after surgery, instead of the day of surgery, and it reaches its maximum intensity point between 12 and 24 h or on the first postoperative day [21]. The present study found that the highest VAS score was reported 6 h postoperatively and that at seven days, the pain was practically less than 1, almost close to 0.

This study is the first randomized clinical trial to demonstrate that the shoulder movement routine had no additional benefit on postoperative shoulder pain in women who underwent uncomplicated HTL.

Multiple perioperative techniques have been described to reduce postoperative pain, among them the use of CO_2_ pressures no greater than 15 mmHg, as reported in a study in which 42 were subjected to standard insufflation of 15 mmHg and 41 to low insufflation of 8–12 mmHg during laparoscopic procedures, resulting in lower maximum inspiratory pressures and CO_2_ absorption, as well as a significant decrease in postoperative intravenous morphine consumption in the low-pressure group [22]. In another study, the Valsalva maneuvers by the anesthesiologist at the end of surgery reduced residual abdominal gas and shoulder pain in patients after laparoscopic cholecystectomy [23]. Another maneuver is the complete aspiration of air from the abdominal cavity, starting from the pelvic cavity in the Trendelenburg position, followed by placing patients in the Fowler position to allow residual gas to move to the subdiaphragmatic area, where the suction tube, placed next to the chamber channel, extracted the remaining air; it was reported to significantly decrease shoulder pain [21].

The shoulder movements employed in our study were simple isometric movements, which could also qualify as shoulder mobilizations, and bear a resemblance to techniques evaluated in a study assessing Maitland’s passive accessory mobilization, where such mobilization elevated the pressure pain threshold at the trapeziometacarpal joint but did not enhance motor function in patients with thumb carpometacarpal osteoarthritis [24]; however, in our study, we did not find an analgesic outcome. Another study of mobilization for the elbow sought to establish if this intervention could elicit physiological effects akin to those observed in specific spinal manipulation techniques, where noted improvements in pain corresponded with enhanced pain-free grip strength and heightened pressure pain thresholds, contrary to our findings [25].

It has been reported that using deep neuromuscular blockade with a low-pressure pneumoperitoneum of 8 mmHg significantly reduces shoulder pain post-laparoscopic hysterectomy compared to a standard-pressure pneumoperitoneum of 12 mmHg [26]. In our institution, the CO_2_ pressure was maintained between 14 and 15 mmHg, aligning with protocols outlined by other authors [27,28], specifically to the preference of some of the participant surgeons to maintain this pressure to enhance operative visibility and maneuverability while ensuring patient safety. In another study, it was observed that CO₂ pressure critically impacted the clarity of the surgical field during laparoscopic procedures, with optimal visualization achieved exclusively at 15 mmHg; a statistically significant deterioration in visibility was noted when the pressure was reduced from 15 to 10 and from 10 to 5 mmHg [29].

In another study, an examination of the postoperative shoulder, periumbilical, and lower pelvic pain assessed on the day of surgery and on postoperative days 1 through 7, no statistical disparities were observed in pain scores across traditional laparoscopy and the gasless technique [30]. Like them, the VAS values found at seven days are very low, with no statistical difference between our groups.

Our results closely align with those reported in a similar study, where across all patients, the average postoperative shoulder pain scores measured via the visual analog scale were 3.37 ± 2.10 at 6 h and 1.95 ± 1.44 at 24 h, showing a significant reduction in pain intensity over this period [31]. Furthermore, both studies observed that VAS scores dropped to below 0 by 48 h post-surgery, underscoring a common trend where shoulder pain typically peaks at 24 h and subsides rapidly within the first few days following surgery, mirroring our findings of pain resolution.

The use of drains has also been controversial regarding its results in terms of postoperative pain. A study found that the group with abdominal drainage showed significantly higher pain scores compared to patients without drainage; this same use prolonged the duration of surgery and postoperative hospital stay; furthermore, it was discovered that the utilization of abdominal drains is related to wound infection [32]. Our results only demonstrated that shoulder pain was 0 in the negative pressure drain group at seven days, with a *p*-value of 0.003. However, the pain was very low in both groups, probably without clinical relevance.

The mechanism of shoulder pain is multifactorial, but in a study of post-thoracotomy patients, the experimental group underwent physiotherapy interventions that consisted of progressive shoulder exercises supervised daily until discharge and received a postoperative exercise and movement booklet upon being discharged; this group had 1.3 units less shoulder pain [33]. Other studies sought a multimodal treatment to improve postoperative pain with measures that are easily achievable in the postoperative period [34]. Another recently applied protocol that modifies the VAS at 6 and 12 h after surgery is the ERAS (Enhanced Recovery After Surgery) protocol; this protocol includes a series of measures that include early ambulation, encourages movement, and is a series of multimodal interventions to reduce hospital stay and perioperative complications [35]. Like these studies, we believe all the maneuvers described need to be performed to minimize shoulder pain [3]. In another study, both massage therapy and TENS significantly reduced PLPS intensity compared to the control group, with no significant difference between the two treatments, demonstrating their effectiveness in managing shoulder pain [36]. It remains imperative to continue exploring a technique or sequence of maneuvers that, when applied synergistically, have the potential to alleviate shoulder pain following laparoscopic procedures, and we posit that there is a significant window of opportunity for research in this domain.

In terms of the study’s robustness, its methodological design and the inclusion of a control group are notable strengths. If well, the control group did not execute a shoulder movement routine, but the participants executed a hand movement routine it could be a limitation of the study. Other limitations are present, including a modest cohort size and the lack of direct oversight during the movement routine, which relied on patients’ adherence to instructions and self-reporting, as well as the potential bias introduced by the use of the VAS for pain evaluation, as it may vary depending on individual participant perception and the medical care provided by each team.

Another major area for improvement is that it is a single-center study, which constrains the generalization of the results. For a more definitive conclusion, subsequent research should be directed toward multicenter randomized clinical trials with expanded sample sizes to substantiate these findings. Such studies would offer a more comprehensive understanding and could decisively influence clinical practices for managing postoperative shoulder pain following TLH.

The significance of our study lies in its innovative approach to addressing postoperative shoulder pain through a targeted shoulder movement routine in patients undergoing TLH. This is the first RCT to explore this intervention, establishing a novel link between physical therapy and pain management in this patient population. Although the study did not show a significant reduction in shoulder pain, the well-documented hypoalgesic effects of exercise suggest that more structured routines could be evaluated in future research as an adjunct to analgesics. A limitation of this study is the subjective nature of pain assessment using the visual analog scale (VAS), which is prone to over- or underestimation by patients. Future studies should consider including patient satisfaction as a key outcome measure to provide insights into the overall effectiveness and acceptability of the interventions, thereby enhancing patient-centered care.

## 5. Conclusions

This randomized clinical trial found that the shoulder movement routine did not significantly reduce postoperative shoulder pain in women undergoing uncomplicated TLH. While the intervention did not achieve the desired outcomes, the study provides valuable insights into non-pharmacological pain management approaches in laparoscopic surgery. Future research should consider larger, multicentric trials and include both objective pain assessments and patient satisfaction measures to better understand the effectiveness of such interventions. Continued exploration of combined perioperative techniques may be necessary to effectively address postoperative shoulder pain.

## Figures and Tables

**Table 1:** Clinical and demographic characteristics of women undergoing uncomplicated total laparoscopic hysterectomy according to the study group.

Characteristic	Group 1. Shoulder Movement Routine (*n* = 36)	Group 2. Control Group, Hand Movement Routine (*n* = 39)	*p*
Age (years)	41.5 (38–47)	41 (38–47)	0.96 *
Body mass index (kg/m^2^)	29.7 (25.9–31.1)	28.4 (26.3–34.4)	0.52 *
Duration of surgery (minutes)	130 (90–180)	128 (100–163)	0.79 *
CO_2_ pressure (mmHg)	15 (14–15)	15 (14–15)	0.44 *
Bleeding (mL)	150 (100–200)	100 (50–200)	0.50 *
Number of trocars	4 (3–4)	4 (3–4)	0.82 *
Postoperative in-hospital stay (hours)	24 (21–28)	23 (21–26)	0.08 *
Comorbidities	13 (33%)	8 (22.2%)	0.28 **
Preoperative hemoglobin	12.1 (11.2–12.8)	11.9 (11.1–12.9)	0.95 *
Systolic blood pressure	117(113–119)	120 (116–122)	0.25 *
Diastolic blood pressure	80 (70–85)	80 (70–80)	0.81 *
Heart rate	78 (70–84)	75 (72–82)	0.40 *
Respiratory rate	19 (18–22)	20 (18–22)	0.60 *
Chronic pain syndrome	1 (2.6%)	1 (2.8%)	0.55 ^&^
Use of rescue analgesia	0 (0%)	2 (5.6%)	0.49 ^&^
Use of postsurgical drainage	8 (20.5%)	48(22.2%)	0.85 **

Values are expressed as median and (interquartile range) or frequency and percentage. * Mann–Whitney U test or ** chi-square test. ^&^ Fisher’s exact test. CO_2_, carbon dioxide.

**Table 2:** Comparison of pain in visual analog scale between the study groups.

Characteristic	Group 1. Shoulder Movement Routine (*n* = 36)	Group 2. Control Group, Hand Movement Routine (*n* = 39)	*p* *
Routines performed	9 (8–11)	9 (7–10)	0.78 *
Shoulder pain at 0 h	0 (0–0)	0 (0–0)	0.99 *
Shoulder pain at 6 h	3 (2–5)	4 (1–5)	0.57 *
Shoulder pain at 24 h	1 (0–4)	2 (0–4)	0.69 *
Shoulder pain at 7 days	0 (0–0)	0 (0–0)	0.91 *
Incision pain at 6 h	4 (3–6)	5 (3–6)	0.73 *
Incision pain at 24 h	3 (2–4)	3 (2–4)	0.94 *
Incision pain at 7 days	0 (0–0)	0 (0–0)	0.92 *
Abdominal pain at 6 h	4 (3–5)	4 (3–5)	0.83 *
Abdominal pain at 24 h	3 (2–4)	2 (1–3)	0.28 *
Abdominal pain at 7 days	0 (0–0)	0 (0–0)	0.41 *

Values expressed as median and (interquartile range). * Mann–Whitney U test. h = hours.

**Table 3:** Comparison of pain in visual analog scale between different times in shoulder movement group.

*n* = 36	Pain at 6 h	Pain at 24 h	Pain at 7 Days	6 h to 24 h *p* *	6 h to 7 Days *p* *	24 h to 7 Days *p* *
Shoulder Pain	3 (2–5)	1 (0–4)	0 (0–0)	0.001	0.001	0.001
Incisional Pain	4 (3–6)	3 (2–4)	0 (0–0)	0.001	0.001	0.001
Abdominal Pain	4 (3–5)	3 (2–4)	0 (0–0)	0.001	0.001	0.001

Values expressed as median and (interquartile range). * Friedman test with post hoc Wilcoxon. h = hours.

**Table 4:** Comparison of pain in visual analog scale between different times in control group (hand movement routine).

*n* = 39	Pain at 6 h	Pain at 24 h	Pain at 7 Days	6 h to 24 h *p* *	6 h to 7 Days *p* *	24 h to 7 Days *p* *
Shoulder Pain	4 (1–5)	2 (0–4)	0 (0–0)	0.001	0.001	0.001
Incisional Pain	5 (3–6)	3 (2–4)	0 (0–0)	0.001	0.001	0.001
Abdominal Pain	4 (3–5)	2 (1–4)	0 (0–0)	0.001	0.001	0.001

Values expressed as median and (interquartile range). * Friedman test with post hoc Wilcoxon. h = hours.

## Data Availability

Please contact the corresponding author for data requests.
